# Determination of the Prevalence of Barr Body in Buccal Smear in the East Khasi Hills Population

**DOI:** 10.7759/cureus.112952

**Published:** 2026-07-19

**Authors:** Kishanth S, Amar J Patowary, Lokesh Ravi V Naidu, Daunipaia Slong, Amarantha D Ropmay, Prabal Das

**Affiliations:** 1 Forensic Medicine and Toxicology, All India Institute of Medical Sciences, Rishikesh, IND; 2 Forensic Medicine, North Eastern Indira Gandhi Regional Institute of Health and Medical Sciences, Shillong, IND

**Keywords:** barr bodies, disaster victim identification (dvi), forensic identification, sex determination, sex determination forensic medicine

## Abstract

Purpose

Sex determination is an important identification data of an individual. Among various methods of sex determination, nuclear sexing methods hold pristine value, as it can be done in cases of mutilated remains and in cases of decomposition. X chromatin is inactivated naturally and is seen in the nucleus as Barr bodies. Buccal mucosa is an ideal sample as it is non-invasive. The study was done to estimate the prevalence of Barr bodies among the tribal population of Meghalaya, India.

Method

Buccal mucosa samples were collected by gentle scraping from both sides of the mouth, smeared on a slide, subjected to Hematoxylin and Eosin staining; 100 cells were viewed under a microscope, data was collected, and appropriate statistical tests were applied.

Results

A total of 171 participants were included, comprising 91 (53.2%) males and 80 (46.8%) females. Barr bodies were identifie in 80/80 (100%) females and 4/91 (4.4%) males. The mean Barr body count was significantly higher among females (43.05 ± 7.00) than males (0.08 ± 0.45; p < 0.001). No significant association was observed between the interval from sample collection to laboratory processing and Barr body detection (p = 0.465).

Conclusion

The percentage of Barr bodies among men and women differs significantly, and the distribution is statistically significant. Although Barr body positivity was observed in four male participants, cytogenetic confirmation by karyotyping or fluorescence in situ hybridization (FISH) was not performed; therefore, these findings cannot be interpreted as evidence of underlying sex chromosome abnormalities. There was no effect on detection upon the time difference between sample collection and processing, so the samples can be collected in an outside setting and then processed in a lab.

## Introduction

Identification of an individual is a fundamental objective in forensic medicine and plays a pivotal role in both criminal investigations and civil proceedings. Establishing the biological profile of unknown human remains begins with determining sex, as it significantly narrows the pool of possible identities. While sex can usually be determined by examining external genitalia and secondary sexual characteristics, these methods become unreliable in cases involving severely mutilated, charred, skeletonized, or decomposed remains. Under such circumstances, nuclear sex determination using sex chromatin offers a valuable alternative. Barr bodies are condensed, transcriptionally inactive X chromosomes present within the nuclei of somatic cells. Because females possess two X chromosomes, one undergoes lyonization during early embryonic development, resulting in the formation of a Barr body [1]. Consequently, Barr bodies are routinely identified in approximately 20-30% of buccal epithelial cells in healthy females, whereas they are generally absent or observed only occasionally (0-4%) in healthy males [2].

Barr bodies can be demonstrated in several tissues, including buccal mucosa, hair follicles, dental pulp, bone marrow, and vaginal epithelium. Among these, exfoliated buccal mucosal epithelial cells are particularly advantageous because they are readily accessible, non-invasive to obtain, inexpensive, and suitable for large-scale population studies. Furthermore, buccal epithelial cells may also be recovered from salivary stains deposited on high-contact objects such as drinking glasses, cups, cigarette butts, and eating utensils, highlighting their forensic relevance in criminal investigations [3,4].

Although Barr body analysis has been extensively investigated in different populations, to the best of our knowledge, this is the first study evaluating the prevalence and distribution of Barr bodies in buccal mucosal epithelial cells among the tribal population of East Khasi Hills, Meghalaya. Ethnic and population-based variations may influence cytological findings; therefore, establishing regional baseline data is important for forensic applications.

## Materials and methods

Study design and setting

A hospital-based observational cross-sectional study was conducted in the Department of Forensic Medicine after obtaining approval from the Institutional Ethics Committee (Reference No. NEIGR/IEC/M14/T10/2021). The study adhered to the principles of the Declaration of Helsinki. Written informed consent was obtained from all participants before enrolment. The sample size was estimated using OpenEpi Version 3.01. As published prevalence estimates applicable to the target population were unavailable, the calculation was based on a previously reported proportion (12.65%) of female participants demonstrating the highest Barr body frequency category. Using a 95% confidence level and an absolute precision of 5%, the minimum required sample size was calculated to be 170 participants. Accordingly, 171 participants were enrolled in the present study. The study included healthy adults aged 20-60 years, accompanying patients to the hospital who were permanent residents of East Khasi Hills, Meghalaya, and who voluntarily provided written informed consent. Individuals with disorders of sex development (intersex conditions), a history of breast carcinoma, or any condition likely to influence Barr body expression were excluded from the study. 

Sample collection

Participants were instructed to rinse their mouths thoroughly with water before sample collection. Exfoliated buccal epithelial cells were obtained by gently scraping the inner surface of both cheeks using a sterile wooden spatula. The collected material was immediately smeared onto clean glass slides and fixed in 95% ethanol.

Staining procedure

**Figure 1:**
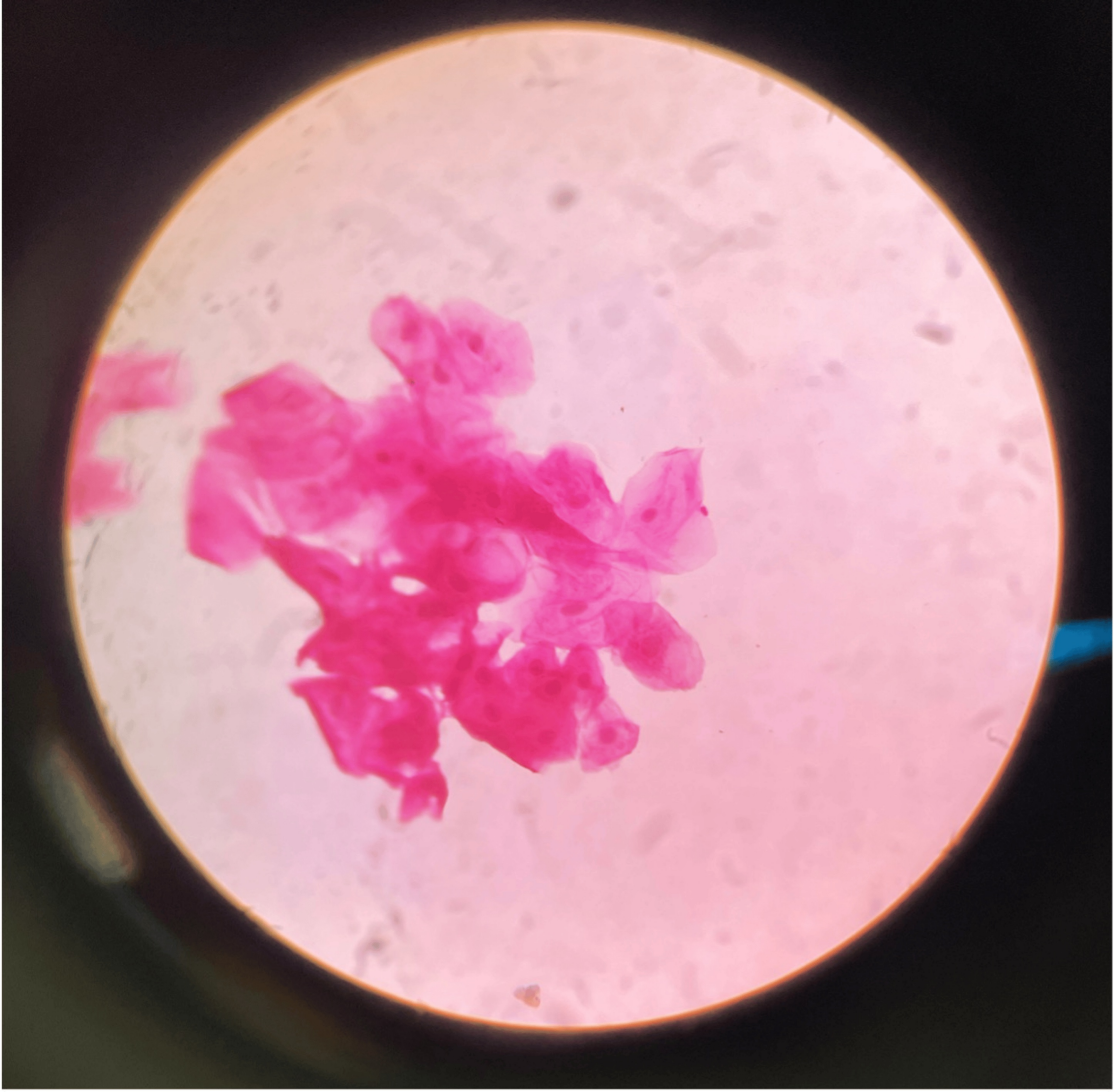
Microscopic view of buccal mucosal cells in the field under 100X magnification

The fixed smears were stained using the hematoxylin and eosin (H&E) technique following standard histopathological procedures. Briefly, slides were stained with Harris hematoxylin, differentiated using 1% acid alcohol, counterstained with 1% aqueous eosin, dehydrated through graded alcohols, cleared in xylene, and mounted with dibutylphthalate polystyrene xylene (DPX) [5-7]. The buccal mucosal cells appear as seen in Figure 1.

Microscopic examination

**Figure 2:**
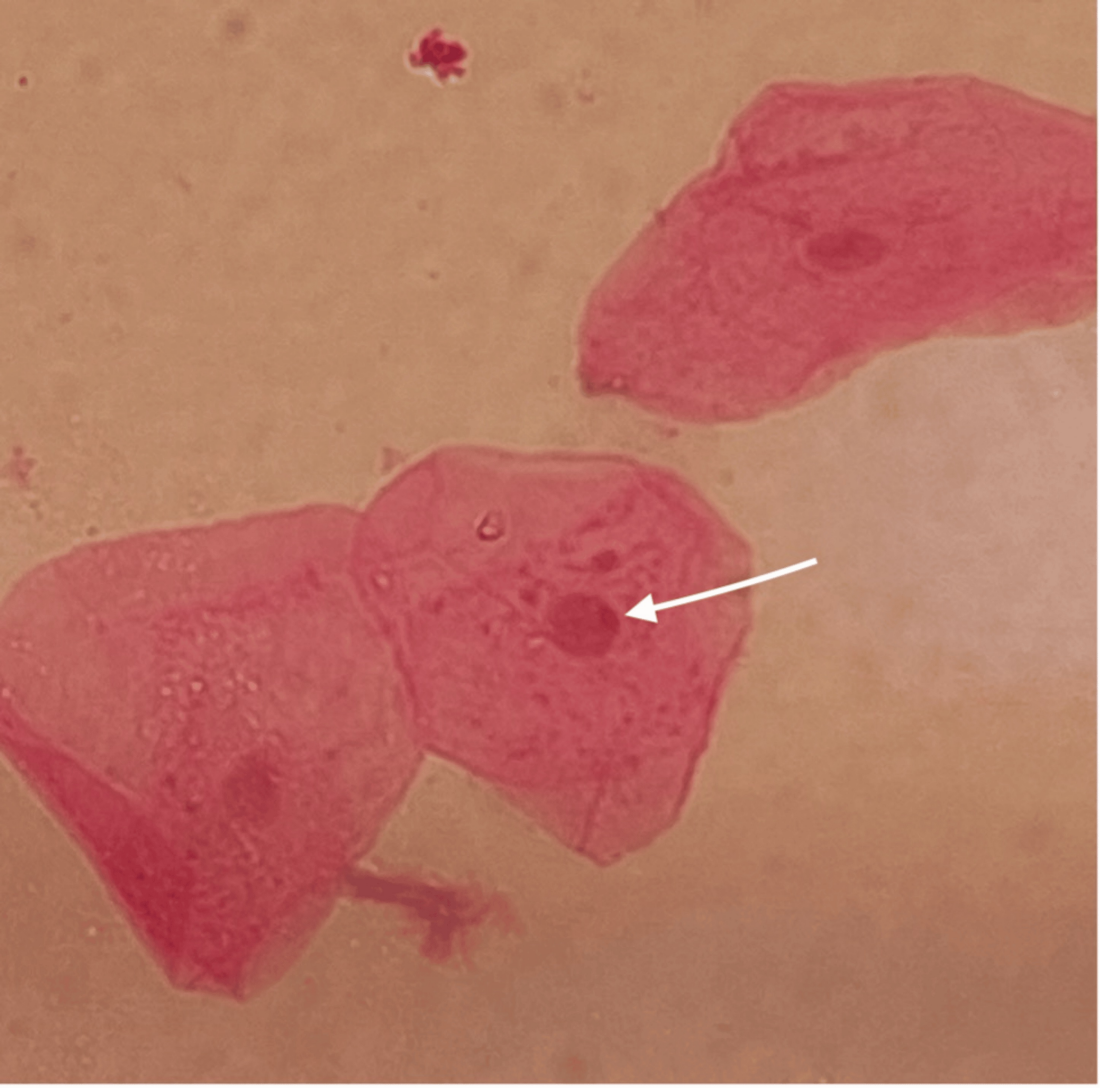
Barr bodies are observed as a plano-convex dark structure seen in the nuclear membrane (arrow pointing to the Barr body)

Each stained smear was examined under a compound light microscope (Olympus CX23) using ×10, ×40, and ×100 (oil immersion) objectives. Routine laboratory calibration and maintenance of the microscope were performed in accordance with institutional protocols. The same immersion oil routinely used in the departmental laboratory was employed for all observations under the ×100 objective. A total of 100 well-preserved, non-overlapping epithelial cells were evaluated per smear. Barr bodies were identified as well-defined, darkly stained plano-convex chromatin masses located adjacent to the nuclear membrane, as seen in Figure 2. Cells that were folded, distorted, or overlapping were excluded from analysis. All slides were examined by a single observer using predefined morphological criteria and were confirmed by a second observer.

Data collected included participants' age, sex, place of residence, time of sample collection, time of laboratory processing, and the number of Barr bodies identified per 100 buccal epithelial cells. Data were entered into Excel (Microsoft, Redmond, Washington) and analyzed using SPSS Statistics version 21.0 (IBM Inc., Armonk, NY, USA). Continuous variables were expressed as mean ± standard deviation (SD) or median (interquartile range (IQR)), whereas categorical variables were presented as frequency and percentage. Differences in mean Barr body counts between males and females were compared using the independent Student's t-test. The association between sex and Barr body positivity was evaluated using the chi-squared test, while the Mann-Whitney U test was used to determine whether the interval between sample collection and laboratory processing influenced Barr body detection. A two-tailed p-value <0.05 was considered statistically significant

## Results

A total of 171 participants were enrolled in the study, comprising 91 males (53.2%) and 80 females (46.8%). Participants ranged in age from 20 to 60 years, with the majority belonging to the 20-29-year age group (138 participants, 80.7%). The remaining participants were aged 30-39 years (20, 11.7%), 40-49 years (11, 6.4%), and ≥50 years (2, 1.2%).

Distribution of Barr bodies according to sex

Barr bodies were detected in all female participants (100%), whereas only four male participants (4.4%) demonstrated Barr body positivity (Table 1).

**Table 1:** Distribution of Barr body based on gender of study participants (N=171)

Gender	Total	Number of subjects showing presence of Barr body, n (%)	Number of subjects showing absence of Barr body, n (%)
Male	91	4 (4.4)	87 (95.6)
Female	80	80 (100)	0 (0)
Total	171	84	87

The normality of Barr body counts was assessed using the Shapiro-Wilk test. The distribution among male participants significantly deviated from normality (W = 0.164, p < 0.001), whereas the distribution among female participants did not significantly deviate from normality (W = 0.979, p = 0.213). Female participants demonstrated significantly higher Barr body counts than male participants (median (IQR): 43 (38-48) vs. 0 (0-0); Mann-Whitney U = 0.0, p < 0.001) (Table 2)

**Table 2:** Comparison of Barr body counts between male and female participants using the Mann-Whitney U test *Data are presented as median (interquartile range), mean ± standard deviation (SD), and range. The distribution of Barr body counts was assessed using the Shapiro-Wilk test. Because normality was violated in the male group, comparisons between males and females were performed using the Mann-Whitney U test. Statistically significant at p < 0.05.

Variable	Males (n = 91)	Females (n = 80)	Mann–Whitney U	p-value
Median (IQR)	0 (0-0)	43 (38-48)	0	<0.001*
Mean ± SD	0.08 ± 0.45	43.13 ± 6.92		
Range	0-4	30-59		

The difference in Barr body prevalence between males and females was highly statistically significant (χ² = 155.69, p < 0.001) (Table 3).

**Table 3:** Association between gender and Barr body detection among study participants (N=171) *Data are presented as n (%). The association between gender and Barr body detection was evaluated using the chi-squared test. Statistically significant at p < 0.05.

Test characteristic	Male n (%)	Female n (%)	Chi-squared value (p-value)*
Barr body present	4 (4.4)	80 (100.0)	155.69 (0.0001)
Barr body absent	87 (95.6)	0 (0)	

Barr body frequency

Among male participants, the number of Barr bodies ranged from 0 to 4 per 100 epithelial cells, with a mean of 0.08 ± 0.45 (Table 4). In contrast, female participants demonstrated Barr body counts ranging from 30 to 59 per 100 epithelial cells, with a mean of 43.05 ± 7.00 (Table 5).

**Table 4:** Descriptive statistics on frequency of Barr bodies among males

Range	Min	Max	Mean	Median	SD
4	0	4	0.08	0.0	0.453

**Table 5:** Descriptive statistics on frequency of Barr bodies among females

Range	Min	Max	Mean	Median	SD
29	30	59	43.05	42.0	7.004

Age-wise distribution

Among males, Barr body positivity was observed only in the 20-29-year age group, where 4 of 74 participants (5.4%) showed detectable Barr bodies. No Barr bodies were identified among male participants aged 30 years or older (Table 6).

**Table 6:** Distribution of Barr body among men based on age

Age interval (years)	Total Subjects (n)	Presence of Barr body n (%)
20-29	74	4 (5.4)
30-39	12	0
40-49	5	0
≥ 50	0	-
Total	91	

Among females, Barr bodies were identified in all participants irrespective of age, with 100% positivity observed across every age group evaluated (Table 7).

**Table 7:** Distribution of Barr body among women based on age

Age interval (years)	Total Subjects (n)	Presence of Barr body (%)
20-29	64	64 (100.0)
30-39	8	8 (100.0)
40-49	6	6 (100.0)
≥ 50	2	2 (100.0)
Total	80	

Effect of sample processing interval

The median interval between sample collection and laboratory processing was 17 hours (interquartile range: 2-18 hours) in both sexes. The interval between sample collection and processing showed no statistically significant association with Barr body detection (Mann-Whitney U test, p = 0.465) (Table 8).

**Table 8:** Testing effect of time difference in samples processing and detection of Barr body

Gender	N	Median	Interquartile range in hours	p-value*
Male	91	17	2-18	0.465
Female	80	17	2-18	

## Discussion

Barr body analysis remains a simple and reliable cytological technique for sex determination, particularly when conventional methods are not feasible. Various biological specimens, including hair roots, dental pulp, and buccal mucosal epithelial cells, have been employed for Barr body detection [8-24]. Among these, exfoliated buccal mucosal cells offer distinct advantages owing to their ease of collection, non-invasive nature, cost-effectiveness, and high participant acceptance. Furthermore, buccal epithelial cells can be recovered from salivary stains on objects frequently encountered at crime scenes, such as drinking glasses, cigarette butts, and utensils, thereby enhancing their forensic applicability.

Several staining techniques have been described for Barr body identification, including hematoxylin and eosin (H&E), Papanicolaou, acridine orange, aceto-orcein, and acriflavine-Schiff stains [14,21,25-27]. Comparative studies have demonstrated variable efficacy among these methods. Priyadharshini et al. reported approximately 97% accuracy with the Papanicolaou stain, whereas Sahana and Ashok found aceto-orcein to provide superior staining quality [26,28]. Baby et al. further demonstrated that acriflavine-Schiff staining yielded better visualization than the Papanicolaou technique [26]. Despite these findings, H&E staining was selected for the present study because it is simple, economical, widely available, and routinely used in histopathology laboratories. The satisfactory results obtained in the present study indicate that H&E staining remains a practical option for Barr body analysis, particularly in resource-limited settings.

The present study demonstrated Barr body positivity in all female participants, whereas only 4.4% of male participants showed detectable Barr bodies. These findings are consistent with those reported by Reddy et al., who also observed universal Barr body positivity among females [1]. Comparable positivity rates have been reported by Jethva et al. (97.47%), whereas Kaur et al. reported a lower prevalence of 76% among females [29,30]. Among males, Galdames et al. and Dixon and Torr reported complete absence of Barr bodies, whereas Jethva et al. and Kaur et al. observed positivity rates of 35% and 16%, respectively [15,17,29,30]. The detection of Barr bodies in 4 of 91 (4.4%) male participants represents an unexpected finding that warrants careful interpretation. Barr body positivity was identified in four of 91 (4.4%) male participants. Although Barr body positivity has been reported in individuals with Klinefelter syndrome (47, XXY) and certain chromosomal mosaicisms, the prevalence observed in the present study is substantially higher than the expected population prevalence of these conditions. Consequently, it is unlikely that all four participants represented true chromosomal abnormalities. Alternative explanations include technical artefacts, staining artefacts, chromocentres, observer-related variability, or other cytological factors that may mimic Barr bodies. As cytogenetic confirmation by karyotyping or fluorescence in situ hybridization (FISH) was not performed, these findings cannot be interpreted as definitive evidence of sex chromosome abnormalities. As cytogenetic investigations, including karyotyping and fluorescence in situ hybridization (FISH), were beyond the scope of the present study, it was not possible to determine whether the observed findings represented technical artefacts or true chromosomal abnormalities. Consequently, the four male participants with detectable Barr bodies should not be regarded as confirmed cases of chromosomal abnormalities. Future studies incorporating repeat smear examination together with cytogenetic and molecular investigations are required to establish the underlying cause of such atypical findings. 

The marked difference in Barr body prevalence between males and females is explained by the phenomenon of X-chromosome inactivation (lyonization). The biological basis of Barr body formation is explained by the Lyon hypothesis, proposed by Mary Lyon in 1961, which states that one of the two X chromosomes in female somatic cells undergoes random inactivation during early embryonic development to achieve dosage compensation between males (XY) and females (XX). X-chromosome inactivation is initiated by expression of the X-inactive specific transcript (XIST) gene, which produces a long non-coding RNA that coats the X chromosome selected for inactivation. This process recruits epigenetic modifications, including DNA methylation, histone hypoacetylation, and histone H3 lysine-27 trimethylation, resulting in chromatin condensation and transcriptional silencing. Microscopically, this condensed inactive X chromosome appears as the Barr body. Although X-chromosome inactivation is generally stable, the proportion of cells exhibiting a visible Barr body may vary because of differences in chromatin condensation, cell cycle stage, epithelial cell maturation, and technical factors affecting cytological preparation and staining. This biological mechanism explains the significantly higher mean Barr body count observed among females (43.05 ± 7.00) compared with males (0.08 ± 0.45) in the present study (p < 0.001). Likewise, the association between sex and Barr body positivity was highly significant (χ² = 155.69, p < 0.001), supporting the utility of Barr body analysis as an adjunctive method for sex determination.

The Barr body counts observed in the present study were comparable to those reported previously. Male participants demonstrated Barr body counts ranging from 0 to 4 per 100 epithelial cells, similar to the findings of Mittal et al. and Jethva et al., although Reddy et al. reported a slightly higher mean count [23,29,3]. Female participants demonstrated Barr body counts ranging from 30 to 59 per 100 epithelial cells, which is consistent with the observations of Mittal et al. and Jethva et al. [23,29]. Collectively, these findings support the reproducibility of Barr body estimation using exfoliated buccal epithelial cells.

Differences in Barr body frequency reported across studies may reflect variations in ethnic and genetic background, staining methodology, sample preparation, selection of epithelial cells for microscopic examination, observer variability, and laboratory protocols. In addition, pathological conditions affecting X-chromosome regulation or chromosomal abnormalities may alter Barr body expression and contribute to discrepancies reported in the literature.

In the present study, Barr bodies were detected in all female participants across the included age groups, and no statistically significant age-related differences were observed. However, this finding should be interpreted with caution because the study population was predominantly composed of young adults (20-29 years), whereas only a small number of participants belonged to the older age groups, particularly those aged 50 years and above. Consequently, the study had limited statistical power to evaluate the influence of age on Barr body expression. Previous studies have reported conflicting observations, with some demonstrating relatively stable Barr body expression throughout adulthood and others suggesting age-related variation. A study involving neonates suggested that Barr body detection is largely independent of age [31]. Aimakhu and Kadiri reported Barr bodies in all female newborns and none in male newborns, whereas Roy et al. observed a transient increase in Barr body frequency during the first few postnatal days [19,22]. In contrast, Sahu et al. suggested that Barr body frequency increases until approximately 30 years of age before declining thereafter [32]. Although these observations indicate possible age-related variation, current evidence remains inconclusive and warrants further investigation in larger, ethnically diverse populations.

An additional objective of this study was to determine whether the interval between sample collection and laboratory processing influenced Barr body detection. No statistically significant association was observed (p = 0.465), indicating that delayed processing within the study conditions did not adversely affect Barr body identification. This finding has important practical implications, as buccal smears collected in field settings or at crime scenes may be transported to the laboratory before processing without substantially compromising the reliability of Barr body detection.

The findings of the present study support the continued use of Barr body analysis as a rapid, inexpensive, and minimally invasive adjunct for sex determination. Although molecular techniques such as DNA profiling remain the gold standard, they may not always be readily available because of financial, technical, or logistical constraints. In such situations, Barr body analysis provides a useful preliminary screening method that can assist forensic experts in narrowing the biological profile of an unknown individual before confirmatory investigations are undertaken.

The present findings reinforce the value of Barr body analysis as a rapid, inexpensive, and minimally invasive adjunct for sex determination. In forensic investigations, exfoliated buccal epithelial cells may be recovered from salivary traces deposited on drinking glasses, bottles, cigarette butts, envelopes, bite marks, chewing gum, and other frequently handled objects. This technique relies on identifying the inactive X chromosome and therefore cannot reliably characterize individuals with certain sex chromosome abnormalities or disorders of sex development. Individuals with Turner syndrome (45, X) typically lack Barr bodies, whereas those with Klinefelter syndrome (47, XXY) may demonstrate Barr body positivity despite being phenotypic males. Similarly, individuals with complete androgen insensitivity syndrome possess an XY karyotype but usually do not exhibit Barr bodies despite having a female phenotype. Chromosomal mosaicism and other disorders of sex development may also produce atypical or misleading Barr body patterns, limiting the diagnostic accuracy of cytological evaluation alone. Consequently, Barr body analysis should always be interpreted in conjunction with clinical findings and, where indicated, confirmed by cytogenetic or molecular investigations.

Limitations 

The present study has certain limitations. It was conducted at a single tertiary care center with a relatively modest sample size, which may limit the generalisability of the findings. The study population was restricted to the East Khasi Hills district of Meghalaya; therefore, the findings may not be directly applicable to other ethnic populations. The age distribution of the study population was uneven, with the majority of participants belonging to the 20-29-year age group. Consequently, the study had limited power to evaluate age-related differences in Barr body expression, particularly among older adults. In the present study, microscopic examination was performed by one observer and subsequently reviewed by a second observer to confirm the findings; however, independent blinded observations were not recorded. Consequently, statistical measures of interobserver agreement, such as Cohen's kappa or the intraclass correlation coefficient (ICC), could not be calculated retrospectively. Future studies should focus on this agreement, as Barr body observation is highly observer-dependent. Furthermore, cytogenetic confirmation by karyotyping or fluorescence in situ hybridization (FISH) was not performed for the four male participants demonstrating Barr body positivity. Consequently, the observed findings cannot be attributed to underlying sex chromosome abnormalities and may instead represent technical artefacts or observer-related variability.

## Conclusions

Barr body analysis using exfoliated buccal mucosal epithelial cells is a simple, economical, and minimally invasive adjunctive technique for preliminary biological sex estimation. The present study demonstrated significant differences in Barr body prevalence and frequency between male and female participants from the East Khasi Hills population. However, Barr body positivity observed in four male participants should be interpreted with caution. As cytogenetic confirmation by karyotyping or fluorescence in situ hybridization (FISH) was not performed, these findings cannot be considered diagnostic of sex chromosome abnormalities and may represent technical or interpretative artefacts. Therefore, Barr body analysis should be regarded as an adjunctive screening method rather than a substitute for cytogenetic or DNA-based investigations, which remain the reference standard for definitive sex determination
